# The Stand-Alone PilZ-Domain Protein MotL Specifically Regulates the Activity of the Secondary Lateral Flagellar System in *Shewanella putrefaciens*

**DOI:** 10.3389/fmicb.2021.668892

**Published:** 2021-06-01

**Authors:** Anna Pecina, Meike Schwan, Vitan Blagotinsek, Tim Rick, Patrick Klüber, Tabea Leonhard, Gert Bange, Kai M. Thormann

**Affiliations:** ^1^Department of Microbiology and Molecular Biology, Justus-Liebig-Universität Gießen, Giessen, Germany; ^2^Department of Chemistry, SYNMIKRO Research Center, Philipps-University Marburg, Marburg, Germany

**Keywords:** flagella, c-di-GMP, flagellar motor, YcgR, *Shewanella*, PilZ domain, lateral flagella

## Abstract

A number of bacterial species control the function of the flagellar motor in response to the levels of the secondary messenger c-di-GMP, which is often mediated by c-di-GMP-binding proteins that act as molecular brakes or clutches to slow the motor rotation. The gammaproteobacterium *Shewanella putrefaciens* possesses two distinct flagellar systems, the primary single polar flagellum and a secondary system with one to five lateral flagellar filaments. Here, we identified a protein, MotL, which specifically regulates the activity of the lateral, but not the polar, flagellar motors in response to the c-di-GMP levels. MotL only consists of a single PilZ domain binding c-di-GMP, which is crucial for its function. Deletion and overproduction analyses revealed that MotL slows down the lateral flagella at elevated levels of c-di-GMP, and may speed up the lateral flagellar-mediated movement at low c-di-GMP concentrations. *In vitro* interaction studies hint at an interaction of MotL with the C-ring of the lateral flagellar motors. This study shows a differential c-di-GMP-dependent regulation of the two flagellar systems in a single species, and implicates that PilZ domain-only proteins can also act as molecular regulators to control the flagella-mediated motility in bacteria.

## Introduction

Numerous species of bacteria in nature are motile by flagella, which allow them to actively move toward more favorable environments. Flagella are long helical proteinaceous filaments, which extend from the cell’s surface and are rotated by the membrane-embedded flagellar motor. The flagellar motor is an intricate nanomachine, which is powered by ion gradients. Most flagella depend on H^+^ gradients, but numerous bacteria employ Na^+^-driven motors (Berg, 2003; Terashima et al., 2017; Nakamura and Minamino, 2019). Torque is created between the stators, transmembrane protein complexes attached to the cell wall, which act as ion-specific channels, and the motor’s C-ring within the cytoplasm. The stators are formed by two proteins, commonly referred to as MotA and MotB in H^+^-dependent motors and PomA and PomB in Na^+^-dependent motors (Kojima, 2015; Lai et al., 2020).

**FIGURE 1:**
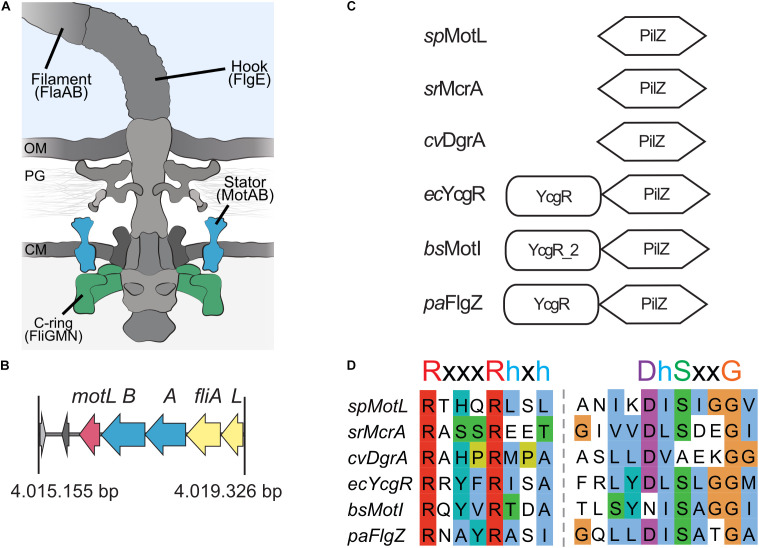
MotL is a putative c-di-GMP-binding PilZ domain-only protein. **(A)** Cartoon of the flagellar motor with the major components (filament, hook, and basal body containing a type III export machinery and the flagellar motor). Torque to drive rotation of the *Shewanella putrefaciens* lateral flagella is created between the stator subunits (proteins MotA and MotB, blue) and the cytoplasmic C-ring (proteins FliG, FliM, and FliN, green). OM, outer membrane; PG, peptidoglycan; CM, cytoplasmic membrane. **(B)** Organization of the *motL* gene region in *S. putrefaciens*, motL is immediately following downstream of the genes *motA* and *motB* that encode the stator subunits. **(C)** Domain organization of PilZ proteins implicated in the regulation of flagella-mediated motility. See text for details. **(D)** Alignment of the PilZ domains of the proteins given in panel **(C)**. The residues critical for c-di-GMP binding are marked above the sequences.

The C-ring is located at the cytoplasmic end of the flagellum and consists of a number of copies of the subunits FliG, located close to the membrane adjacent to the stators, as well as FliM and FliN (Francis et al., 1992, 1994; Zhao et al., 1996). Ion translocation through the stators is thought to result in conformational changes, which are then translated into the rotational movement of the C-ring by electrostatic interaction of the cytoplasmic parts of the stators’ A-subunits with the C-terminal domain of FliG (for a cartoon of the flagellar apparatus, see Figure 1A). Most flagellar motors are bi-directional and allow counterclockwise and clockwise rotations, which are regulated by one or more associated chemotaxis systems to allow a directed movement by means of a so-called random walk (Sourjik and Wingreen, 2012; Bi and Sourjik, 2018).

A fully assembled flagellum consists of numerous proteins, among which the flagellins, which are the major building blocks of the filament, may add up to several 10,000 copies (Nakamura and Minamino, 2019). Depending on the species and motor, the flagellar rotation rate can reach up to more than 100 Hz, and each rotation is accompanied by a massive influx of the corresponding coupling ion (Meister et al., 1987). Thus, the assembly and operation of a flagellum put a substantial burden on the cell’s metabolism, and therefore, under some conditions, such as during biofilm formation or shortage of nutrients, a fast rotation of the motor may not be advantageous. Accordingly, flagella formation and activity are highly regulated, and many bacterial species have developed appropriate systems that, in addition to chemotaxis systems, enable the cells to control the activity of the flagellar motor (Subramanian and Kearns, 2019). Recent studies have demonstrated that some species may even choose to eject major parts of the flagellum when nutrients are lacking (Ferreira et al., 2019; Kaplan et al., 2019; Zhu et al., 2019; Zhuang et al., 2020).

One means to control the activity of the flagellar motors is through the proteins that act as brakes or clutches to slow down the flagellar rotation in response to levels of the secondary messenger molecule c-di-GMP (Brown et al., 2011; Subramanian and Kearns, 2019). Such motor-affecting proteins have been identified in several species, e.g., YcgR in *Escherichia coli* and *Salmonella enterica* (Boehm et al., 2010; Fang and Gomelsky, 2010; Paul et al., 2010), MotI (DgrA) in *Bacillus subtilis* (Chen et al., 2012; Gao et al., 2013; Subramanian et al., 2017), and FlgZ in *Pseudomonas* species (Martinez-Granero et al., 2014; Baker et al., 2016; Wirebrand et al., 2018). All these proteins are characterized by a so-called PilZ domain, which is responsible for binding the effector molecule c-di-GMP upon which the protein directly interacts with the components of the flagellar motor to slow down the rotation. These motor-effector proteins likely function by interfering with normal rotor-stator interactions. In *E. coli*, interaction of YcgR has been mapped to the C-ring proteins FliG and FliM as well as to the stator protein MotA (Boehm et al., 2010; Fang and Gomelsky, 2010; Paul et al., 2010; Hou et al., 2020). MotA has also been demonstrated to be a binding target of *B. subtilis* MotI/DgrA, and similarly for *Pseudomonas* aeruginosa FlgZ, an interaction with an orthologous stator protein, MotC, has been shown (Chen et al., 2012; Baker et al., 2016; Subramanian et al., 2017).

The occurrence of c-di-GMP-dependent motor-regulating proteins in several different and somewhat unrelated bacterial species suggests that they represent a common means for flagellar motor control. However, such functional YcgR-like motor-effector proteins have not yet been described for species belonging to the polarly flagellated gammaproteobacteria of the genus *Vibrio*, *Shewanella*, and others. *Vibrio cholerae* and *Vibrio alginolyticus* possess homologs to YcgR, named PlzD (Pratt et al., 2007; Kojima et al., 2019). *Vc*PlzD has been shown to specifically bind c-di-GMP and the corresponding binding site was identified by crystallization (Benach et al., 2007; Pratt et al., 2007). Overproduction of both *Vc*PlzD and *Va*PlzD in the corresponding species resulted in a negative effect on motility, however, this effect occurred independently of c-di-GMP binding (Pratt et al., 2007; Kojima et al., 2019). So far, it remains unclear by which mechanism *Vibrio* PlzD affects the flagella-mediated motility.

In addition, in *Shewanella* sp., c-di-GMP levels regulate the flagella-mediated motility. In *Shewanella oneidensis* and *S. putrefaciens*, the multidomain transmembrane phosphodiesterase PdeB strongly affects the flagella-mediated swimming in response to the nutrient conditions. Cells lacking *pdeB* exhibit a higher overall c-di-GMP concentration and a drastically decreased spreading through soft agar (Chao et al., 2013; Rossmann et al., 2019). *S. putrefaciens* possesses two distinct flagellar systems, the main Na^+^-dependent polar system, which mediates the main propulsion during free swimming, and screw thread motility through structured environments which is controlled by the chemotaxis system (Bubendorfer et al., 2012, 2014; Kühn et al., 2017, 2018). In addition, depending on the media conditions, a secondary lateral flagellar system can be formed, which is powered by H^+^ ions and assists in the navigation and spreading through structured environments (Bubendorfer et al., 2012, 2014). We found that, although being localized to the flagellated cell pole, PdeB regulates the lateral flagellar system. Part of this effect is explained by a significant decrease in the lateral flagellar gene expression, but it remains unclear if and how an elevated c-di-GMP level affects both flagellar systems also at the posttranscriptional level (Rossmann et al., 2019). We therefore set out to identify the potential c-di-GMP-dependent factors that regulate the motor activity. Here, we identified and characterized a PilZ-domain protein, MotL, which lacks the N-terminal domain present in YcgR, MotI, or FlgZ, and specifically acts on the lateral flagella in dependence of c-di-GMP.

## Results

### Identification of a Flagellar Motor Effector Protein in *Shewanella putrefaciens*

To identify the potential c-di-GMP-dependent effectors in *Shewanella putrefaciens*, we started with a genomic screen for homologs of the previously characterized flagellar regulators. The c-di-GMP-dependent functional regulators of the bacterial flagellar motors studied so far, *E. coli* YcgR, *Pseudomonas* FlgZ, and *B. subtilis* MotI, all possess a characteristic c-di-GMP-binding PilZ domain and an N-terminal domain referred to as the YcgR domain (Cheang et al., 2019). In *S. putrefaciens*, five proteins are annotated as putative PilZ domain-containing proteins (Sputcn32_2813, Sputcn32_2815, Sputcn32_2212, Sputcn32_1553, and Sputcn32_3446). Apart from possessing a putative PilZ domain, none of these proteins shows noticeable similarities to YcgR, FlgZ, or MotI at the protein level, and so far, none of them has been studied in any detail. Notably, the gene Sputcn32_3446 is located directly downstream of the secondary flagellar gene cluster, immediately following *motA* and *motB*, which encodes the proton-dependent stator of the lateral flagellar motor. We therefore hypothesized that this protein may somehow be involved in the formation and/or activity of the lateral and, potentially, the polar flagella.

Sputcn32_3446, annotated as a PilZ domain, is located 34 bp downstream of *motB* and transcribed in the same direction (Figure 1B). The gene is 435 bp in length and encodes a protein of 144 aa with an estimated molecular mass of 16.6 kDa and a theoretical pI of 5.94. The protein is thus much smaller than YcgR (244 aa), FlgZ (263), and MotI (217 aa) as an N-terminal YcgR domain is not present (Figure 1C). The predicted c-di-GMP-binding motifs (**RxxxRhxh, DhSxxG**; Galperin and Chou, 2020) are fully conserved (Figure 1D). The protein is conserved in a number *Shewanella* species that possess dual flagellar systems, and the gene it is always located downstream of *motB*. Potential homologs of Sputcn32_3446 are also present in some species of *Aeromonas* and *Vibrio*, but absent from the well-characterized *V. parahaemolyticus* and *V. alginolyticus*, which also possess two distinct flagellar systems. We henceforth referred to the protein as MotL, relating to its location within the lateral flagellar gene operon and its differences to YcgR, FlgZ, and MotI with respect to the protein sequence and absence of further domains.

### MotL Binds c-di-GMP

**FIGURE 2:**
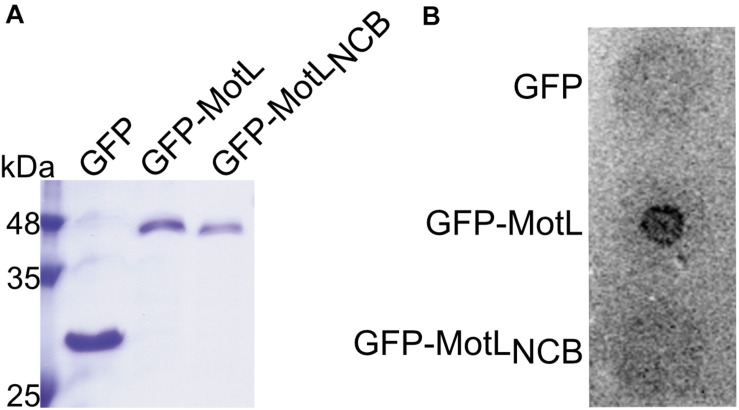
MotL is a c-di-GMP-binding protein. **(A)** Purification of MotL. Shown is a Coomassie-stained SDS gel after separation of His-tagged purified GFP, GFP-MotL, and GFP-MotL_*NCB*_. **(B)** MotL binds c-di-GMP. Purified protein was applied to a membrane as indicated and exposed to radioactively labeled c-di-GMP. Only the MotL with the predicted binding site is able to bind and retain c-di-GMP.

**FIGURE 3:**
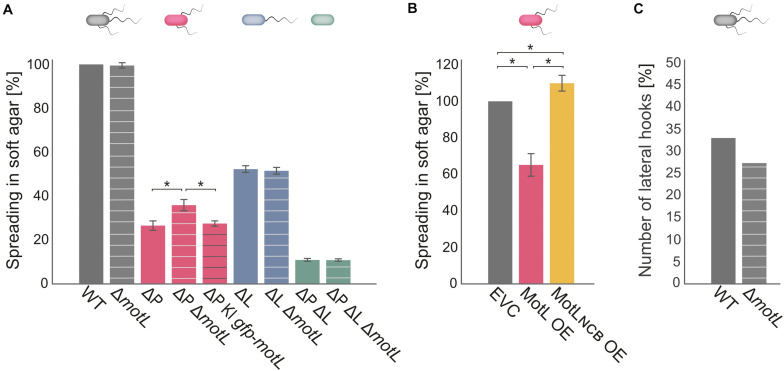
MotL affects the *S. putrefaciens* lateral, but not the polar, flagella. **(A)** Flagella-mediated spreading through soft agar after deletion (and complementation) of *motL.* The corresponding background strains had both the polar and lateral flagella (gray), only one each [ΔP lacking the polar filament (red); ΔL lacking the lateral filament (blue) or no flagella (green) as indicated by the cartoon cells above the bars]. The white cross bands indicate the strains in which *motL* was deleted, black cross bars indicate the strain bearing *gfp-motL*. **(B)** Lateral flagella-mediated spreading upon ectopic overproduction (OE) of GFP-MotL and the corresponding mutant not binding c-di-GMP (GFP-MotL_*NCB*_). EVC, empty-vector control. **(A,B)** are the results of three independent experiments. The bars with the asterisks indicate significant differences according to ANOVA. **(C)** Effect of *motL* deletion on the presence of lateral hooks as a proxy for the number of functional lateral flagella in cells growing exponentially in the LB medium. See text and methods for the hook staining procedure. The numbers are deduced from observing 430 cells for WT and 431 for *motL* deletion in four independent experiments. The difference is not significant according to the *Pearson’s Chi-squared test with Yates’ continuity correction*.

The presence of a potential c-di-GMP-binding site in MotL suggested that MotL function may be governed by c-di-GMP. We therefore determined the ability of MotL to bind c-di-GMP *in vitro*. To this end, we aimed at the overproduction of MotL and a corresponding variant bearing alanine substitutions within all conserved residues in the predicted c-di-GMP binding site (*n*o *c*-di-GMP-*b*inding; MotL_*NCB*_). However, MotL or MotL_*NCB*_ overproduction and purification was only possible when a fusion protein was added to the proteins’ N-termini. For the direct purification from *S. putrefaciens*, we therefore used a variant with a sfGFP fused to the MotL N-terminus (sfGFP-MotL and sfGFP-MotL_*NCB*_) which was produced from a plasmid. This fusion protein could be stably produced and purified (Figure 2A), fully complemented a *motL* deletion mutant (see Figure 3A and Supplementary Figure 1) and, therefore, also allowed later studies on swimming behavior and localization. MotL and MotL_*NCB*_ fusion proteins were purified directly from *S. putrefaciens*, and the binding of radioactively labeled c-di-GMP to both variants was determined. However, under these conditions, no significant c-di-GMP binding to MotL was observed. We hypothesized that c-di-GMP may already be bound to MotL and, therefore, both protein variants were purified from a strain also producing the phosphodiesterase (PDE) PdeH, which has previously been shown to effectively lower the c-di-GMP levels in *S. oneidensis* (Thormann et al., 2006). Produced under these conditions, robust c-di-GMP binding to MotL could be observed. In contrast, the MotL_*NCB*_ variant was unable to bind this second messenger (Figure 2B). Based on these findings, we hypothesized that MotL likely functions in a c-di-GMP-dependent fashion.

### MotL Exclusively Affects Swimming Mediated by the Lateral Flagella

To determine if MotL has an effect on swimming, we deleted the corresponding gene from the chromosome. The resultant mutant cells were then tested for the ability to spread through soft agar as a measure for the ability of flagella-mediated motility (Figure 3). Loss of *motL* had no significant effect on the spreading of the wild-type cells. However, as swimming motility of *S. putrefaciens* through soft agar is synchronously mediated by both the main polar and secondary lateral flagella (Bubendorfer et al., 2012, 2014), potential effects on only one of the systems may be masked. We therefore introduced the *motL* deletion into strains in which the flagellin-encoding genes of the polar (Δ*flaAB*_1_) or lateral (Δ*flaAB*_2_) systems were deleted, so that these strains only produce one type of functional flagella. Spreading of cells with only the polar flagellum through soft agar was not affected in the absence of MotL. In contrast, cells that only possessed the lateral flagella showed a significant increase in spreading, and this effect could be complemented by the reintegration of *motL* or *sfgfp-motL* into its native gene locus (Figure 3A). To determine if the increase in spreading may be caused by an increase in the number of lateral flagella, we introduced the *motL* deletion into a strain in which the lateral flagellar hook structures–as a proxy for an established lateral flagellar system–can be labeled by the coupling of a fluorescent dye to the major hook protein FlgE_2_ (FlgE_2*T*242*C*_). Visualization and quantification of the number of cells with lateral hooks by fluorescent microscopy revealed that the population of flagellated cells slightly decreased in the absence of MotL from about 33 to about 26% (Figure 3C). Thus, the enhanced spreading in soft agar in Δ*motL* mutants is not due to an increase in flagellation. As previous studies showed that the lateral flagellar system is not controlled by the chemotaxis system of *S. putrefaciens* (Bubendorfer et al., 2012, 2014), an effect of MotL on navigation in cells motile by only lateral flagella was also ruled out.

As the deletion of MotL had a positive effect on the spreading of the *S. putrefaciens* cells with lateral flagellar systems, we hypothesized that overproduction may have the opposite effect. We therefore produced (sfGFP)MotL from a plasmid and determined its effect on spreading (Figure 3B). As expected, overproduction of MotL negatively affected the spreading of the wild-type (−15%) and lateral flagella-only cells (−27%), while cells with only the polar flagella exhibited a minor decrease (−4%) in spreading. We have previously observed that the addition of the inducer anhydrotetracycline hydrochloride (AHT) has a negative effect on the flagella-mediated motility due to a yet unknown mechanism (Brenzinger et al., 2018), which may account for the reduction in the spreading seen for the latter strain. We therefore concluded that MotL specifically acts as a regulator on the lateral flagella function.

### MotL Controls the Activity of the Lateral Flagella in Response to c-di-GMP

**FIGURE 4:**
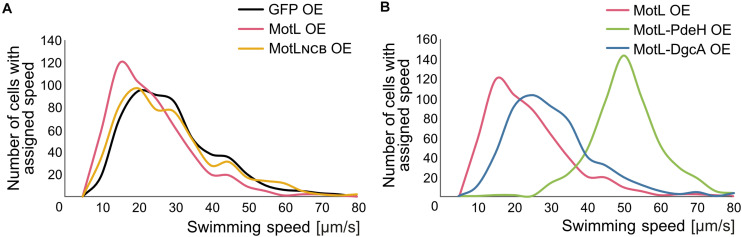
MotL affects the swimming speed mediated by the lateral flagella in a c-di-GMP-dependent fashion. **(A)** Effect of an OE of MotL (red), MotL_*NCB*_, and GFP (control) on the swimming speed of a strain only possessing the lateral flagella (ΔP). **(B)** Effect of an OE of MotL alone (red) or in concert with the phosphodiesterase PdeH (green) or the diguanylate cyclase DgcA (blue). For both **(A,B)** the swimming speeds of 591 cells per strain were analyzed. See also Supplementary Figure 3 for the statistical analysis.

The gain of the lateral flagella-mediated spreading on soft agar upon loss of MotL and the observed c-di-GMP binding by this protein strongly suggested that MotL acts as a c-di-GMP-dependent regulator, such as a brake or clutch, on the lateral flagellar motors. To further investigate this, (GFP)MotL or (GFP)MotL_*NCB*_ were ectopically produced in a *S. putrefaciens* strain only capable of forming the lateral, but not the polar, flagella (Δ*flaAB*_1_). To determine the direct effect of MotL on the performance of the lateral motor, we measured the speed of free-swimming cells upon MotL overproduction by cell tracking using light microscopy (Figure 4). The velocities of a strain harboring the vector control (only producing sfGFP) showed a wide speed distribution from about 10 to about 60 μm s^–1^ with most cells exhibiting a speed between 10 and 40 μm s^–1^ (Figure 4A and Supplementary Figure 3A). For the cells overproducing MotL, the subpopulation of the slow swimming cells (in particular, between 10 and 30 μm s^–1^) increased, and the average speed dropped significantly from 24 to 19 μm s^–1^. In contrast, the speed distribution of cells producing the MotL_*NCB*_ variant was highly similar to that of the control strain, suggesting that c-di-GMP binding is required for MotL function.

In the next set of experiments, we therefore determined the effect of high or low c-di-GMP levels on the role of MotL in regulating the lateral flagella function (Figure 4B). To this end, we produced MotL together with the phosphodiesterase PdeH from *E. coli* or the diguanylate cyclase DgcA (VCA0956) of *Vibrio cholera*. Both proteins have previously been shown to decrease or increase the intracellular level of c-di-GMP in the closely related *S. oneidensis* (Thormann et al., 2006). To first rule out that the effect of the different c-di-GMP levels on swimming by lateral flagella was mainly due to the effects and mechanisms not mediated by MotL (such as differences in the lateral flagellar gene expression), *pdeH* or *dgcA* were first expressed in the laterally-only flagellated *S. putrefaciens* cells, in which *motL* was deleted, and the spreading of the strains in soft agar was determined (Supplementary Figure 3C). Production of PdeH resulted in an increase in spreading of about 30% while DgcA production lowered the spreading to about 80%. In addition, the swimming speeds of the cells were recorded. In contrast to the spreading phenotype, expression of *dgcA* had almost no effect on the swimming speed of the cells compared to those of the cells bearing the empty vector control (Supplementary Figures 2A,B). Expression of *pdeH* resulted in a slight shift toward faster cells, but the increase was rather minor (Supplementary Figures 3A,B). The results show that production of a diguanylate cyclase (DGC) or a PDE has a notable effect on spreading in soft agar while the swimming speed itself is affected rather little. Thus, other factors than MotL also contribute to the lateral flagella-mediated swimming in dependence of c-di-GMP levels, which is particularly evident in structured environments such as soft agar but less so in the performance of the lateral motors.

In a strain co-producing DgcA and (sfGFP)MotL (high levels of c-di-GMP), the average speed and speed distribution of the cells was similar to that of the cells producing sfGFP–MotL alone (24 versus 26 μm s^–1^; Figure 4B; Supplementary Figures 3A,B), while spreading through soft agar was decreased by about 30% (Supplementary Figure 2C). This strongly suggested that under the conditions applied (exponential planktonic growth in complex media), a further increase of the c-di-GMP levels within the cells is not relevant for the activity of MotL for free swimming. Thus, the observed diminished spreading through soft agar caused by a higher c-di-GMP level is unlikely to be directly mediated by MotL. In sharp contrast, the velocity distribution of the cells at low c-di-GMP levels (MotL co-produced with PdeH) was drastically shifted toward a higher speed (Figure 4B and Supplementary Figures 3A,B) with an average speed of 50 μm s^–1^. In addition, the spreading in soft agar doubled compared to that of the strain expressing MotL alone (Supplementary Figure 2C). Taken together, the data strongly indicate that MotL specifically regulates the activity of the lateral flagellar in response to low c-di-GMP levels.

### MotL Does Not Affect the Lateral Motor Function via the FliL Levels

**FIGURE 5:**
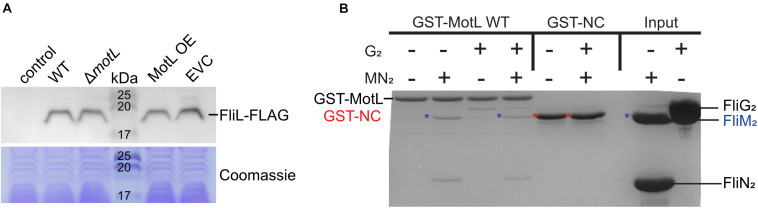
Effect of MotL on FliL levels and MotL interaction with the flagellar C-ring. **(A**) Western blot to determine the levels of (FLAG-tagged) FliL in dependence of MotL levels (upper panel) with the Coomassie-stained polyacrylamide gel as loading control in the panel below. Neither deletion nor overproduction of MotL affects FliL levels. **(B**) Coomassie-stained gel of a GST pulldown with an immobilized MotL protein against the components of the lateral flagellar C-ring (proteins FliG_2_, FliM_2_, and FliN_2_). GST alone (GST-NC) served as the negative control. The position of the proteins in the gel is indicated at the sides of the gel, in addition, blue asterisks indicate the position of FliM2 and red asterisks indicate those of GST-NC.

In the following, we aimed at understanding the mechanism by which FliL mediates its function of the lateral flagellar motor. Previously, another stand-alone PilZ-domain protein, DgrA, had been demonstrated to affect the flagella-mediated swimming in *Caulobacter crescentus* (Christen et al., 2007). In this study, the authors hypothesized that DgrA acts via the flagellar motor protein FliL, as upon the overproduction of DgrA, FliL protein levels were significantly decreased. To determine if MotL functions by a similar mechanism, we quantified the effect of MotL levels on those of the lateral flagella FliL_2_. To allow the detection of FliL_2_, the corresponding gene was replaced at its native locus by a mutant version encoding a C-terminal FLAG-tag. Subsequent analysis showed that the tagged version of MotL was stably produced and fully supported the secondary flagella-mediated spreading in soft agar. Absence or overproduction of MotL did not affect the FliL_2_ levels (Figure 5A), suggesting that MotL has a different mode of function as a flagellar regulator.

### MotL May Directly Interact With the Components of the Lateral Flagellar Motors

The *in vivo* function of MotL as a regulator of the lateral flagella function suggested that MotL, as other PilZ-domain flagellar brakes or clutches, directly or indirectly interacts with the components of the lateral flagellar motors. We therefore performed localization studies by fluorescence microscopy to determine whether fluorescently tagged MotL may interact with the flagellar basal bodies or with the freely diffusing MotAB stator units not engaged in the flagellar motor.

To visualize the general localization, we used the strain harboring the sfGFP-MotL-encoding hybrid gene in the chromosome replacing native *motL*. Co-localization with the lateral flagellar components was performed in strains in which the C-ring protein FliM_2_ or the stator component MotB were additionally labeled by mCherry. In addition, the fused MotL protein was overproduced from a plasmid, and/or the cellular levels were increased by the (co-)production of the phosphodiesterase PdeH or the diguanylate cyclase DgcA. However, under no conditions a distinct localization of sfGFP–MotL occurred, and the protein, if present, was always diffusively localized within the cytoplasm (Supplementary Figure 4).

In the next step, we performed studies on the direct interactions between MotL and components of the flagellar motor. To this end, we conducted a bacterial two-hybrid (BACTH) analysis for the potential interactions between MotL and MotL_*NCB*_ on one side, and the C-ring components FliG_2_, FliM_2_, and FliN_2_ and the stator-forming subunits MotA and MotB on the other side (see cartoon of the flagellar motor in Figure 1A). To this end, all above-mentioned proteins were produced in an appropriate *E. coli* strain at high or low levels as N- or C-terminal fusion to the T18 and T25 fragments of an adenylate cyclase from *Bordetella pertussis*, which are non-functional when separate. However, interaction of two fusion proteins leading to a close proximity of the T18 and T25 fragments results in the formation of an active adenylate cyclase and raising the cAMP levels, which can be readily visualized on an appropriate media. In this assay, MotL and MotL_*NCB*_ did not exhibit any interactions with any of the C-ring or stator components (data not shown).

As a third approach to determine a potential interaction of MotL with the flagellar motor, we performed *in vitro* pull-down assays with purified MotL and components of the C-ring of the lateral flagella (Figure 5B). To this end, MotL was produced and purified as an N-terminal glutathione-*S*-transferase (GST) fusion to be used as bait. Under the conditions tested, purified FliG_2_ as well as FliM_2_ and FliN_2_ showed binding to MotL, while GST did not display any binding to these C-ring components, which may potentially hint at a functional interaction between MotL and the flagellar motor.

## Discussion

For several bacterial species, c-di-GMP has been shown to affect the flagella-mediated motility at the level of motor function by c-di-GMP-binding regulator proteins. Here, we show that in *S. putrefaciens*, such a regulator protein specifically tunes the activity of the lateral, but not the polar, flagellar system. This regulator, now referred to as MotL, basically consists of a single PilZ domain, which has previously been shown to be involved in c-di-GMP binding and, which, accordingly occurs in a number of c-di-GMP-dependent effector proteins (Amikam and Galperin, 2006; Cheang et al., 2019).

We showed that MotL binds c-di-GMP and that binding of this second messenger affects the MotL function, suggesting that rising levels of c-di-GMP decrease the flagella rotation. However, when MotL was produced together with the DGC DgcA, the effect on the swimming speed provided by the lateral flagella was surprisingly small. As we have previously shown that DgcA is an active DGC in *Shewanella* (Thormann et al., 2006) and spreading in soft agar was noticeably reduced upon DgcA production (Supplementary Figures 2C,3C), we assume that DgcA actually raises the level of cellular c-di-GMP. This suggests that the level of c-di-GMP in *S. putrefaciens* under the conditions used for the swimming assays was already sufficiently high to achieve the maximum effect of MotL on the flagellar motors. This hypothesis was corroborated by the finding that c-di-GMP binding to MotL *in vitro* was only achieved when the protein was purified from an *S. putrefaciens* strain in which the PDE PdeH was additionally produced. The effect of DgcA production on spreading in soft agar may thus be rather due to other c-di-GMP-mediated processes such as the downregulation of the lateral flagella and activation of adhesion factors (Rossmann et al., 2019). In contrast, we found a huge effect of MotL on the lateral motors at a low c-di-GMP concentration, where the cells moved at an even higher speed as under the same conditions in the absence of MotL and exhibited a massive increase in spreading. This finding suggests that MotL may not only slow down flagellar rotation but may also speed up swimming mediated by the lateral flagella in response to lower c-di-GMP levels. Generally, marine bacteria, among them, *S. putrefaciens*, have been reported to accelerate to a higher speed in the presence of various amino acids (Barbara and Mitchell, 2003). This process, referred to as chemokinesis, enhances the chemotactic precision as shown for *V. alginolyticus* (Son et al., 2016). The underlying mechanism is yet unknown, and it is also unclear if another protein component is required, e.g., for rotor-stator stabilization, and if MotL may play a role in this process.

The mechanism by which MotL affects the lateral flagella motors remains elusive so far. Several of the c-di-GMP-dependent flagellar motor effectors have been studied in more detail, namely YcgR of *E. coli* and *Salmonella*, MotI (formerly YpfA or DgrA) in *B. subtilis*, and FlgZ in *Pseudomonas*. MotI and FlgZ are thought to predominantly or even exclusively function through interactions with stator units to inhibit proper rotor-stator interactions. MotI (YpfA) was shown to interact with the stator subunit MotA in the BACTH analysis, by the co-purification of MotA with an ectopically overproduced MotI (YpfA), identification and analysis of suppressor mutants in MotA, and localization pattern of fluorescently labeled MotI (Chen et al., 2012; Subramanian et al., 2017). Similarly, *P. aeruginosa* FlgZ was demonstrated to interact with the stator subunit MotC by the BACTH analysis and co-precipitation experiments (Baker et al., 2016). MotCD is the stator that is employed by *P. aeruginosa* during swarming motility. No interaction was found with any of the tested C-ring components and, notably, also none with the subunits of the second stator system MotAB, which inhibits swarming and rather promotes free swimming motility (Doyle et al., 2004; Toutain et al., 2005). Also, fluorescence microscopy on the fluorescently labeled FlgZ showed the expected localization to the single polar flagellar motor in swarming cells. Thus, in *P. aeruginosa*, FlgZ regulates swarming motility in dependence of c-di-GMP levels likely by specifically uncoupling the MotCD stator from the motor.

In contrast, flagellar motor regulation by YcgR of *E. coli* and *Salmonella* does involve both the flagellar stators as well as the flagellar C-ring. Förster resonance transfer studies revealed a c-di-GMP-dependent interaction with the MotAB stator and suppressor mutants in MotA were isolated (Boehm et al., 2010). In addition, FliG and FliM could be co-isolated with overproduced YcgR (Fang and Gomelsky, 2010; Paul et al., 2010), and BACTH studies suggested a direct interaction of YcgR with FliG and FliM (Fang and Gomelsky, 2010). Furthermore, mutations in FliG and FliM affected YcgR binding and inhibition of motility, and the localization of YcgR–GFP as punctae was shown to depend on the presence of FliG and FliM (Paul et al., 2010). Notably, the general interaction of YcgR with the flagellar C-ring appears to be independent of c-di-GMP binding (Fang and Gomelsky, 2010; Paul et al., 2010). Accordingly, a recent study provides evidence that YcgR functions at the interface between MotA and FliG to slow down the flagellar rotation (Hou et al., 2020). In addition, to act as the uncoupler of flagellar rotor and stator, YcgR has been proposed to also regulate chemotaxis by affecting the counterclockwise-clockwise bias of the flagellar motor (Girgis et al., 2007; Fang and Gomelsky, 2010; Paul et al., 2010).

Notably, in contrast to YcgR and other motor regulating proteins, MotL consists of only a PilZ domain. Studies on crystal structures of YcgR-like proteins, MotI, PlzD, and PP4397, showed that for these proteins, c-di-GMP binding involves both the PilZ as well as the N-terminal YcgR domains. This leads to an extensive conformational change, which is assumed to enable the functional interaction (Benach et al., 2007; Ko et al., 2010; Subramanian et al., 2017). Accordingly, the N-terminal domain of YcgR has been suggested to be important for the specificity of YcgR–FliG interactions (Fang and Gomelsky, 2010). However, as MotL does not possess such an N-terminal YcgR domain, interaction with its target protein has to occur in a different fashion. Single PilZ-domain proteins, such as MotL, are thought to serve as c-di-GMP adaptors to control target systems (Christen et al., 2007; Cheang et al., 2019). Even in the absence of a YcgR domain, our *in vitro* assays hint at an interaction of *S. putrefaciens* MotL and FliG, FliM, and FliN of the lateral flagella (see Figure 1A), and we thus suggest that MotL functions include an interaction with the C-ring of the lateral flagellar motors, e.g., by directly or indirectly altering the rotor-stator interactions. Although we were unable, so far, to demonstrate the *in vitro* or *in vivo* interactions of MotL and the MotAB stator, we cannot rule out the possibility that the regulatory MotL function includes a (transient) binding to MotA or MotB, e.g., to weaken or to strengthen the rotor-stator interactions. Further studies are required to elucidate if MotL acts on the flagellar motor directly via FliG and/or the MotAB stators upon c-di-GMP binding and if this involved further factors. A direct effect of MotL on chemotaxis is rather unlikely, as previous studies have indicated that the lateral flagellar motors of *S. putrefaciens* are not responding to the chemotaxis system (Bubendorfer et al., 2012, 2014). However, as the lateral flagella affect the turning angles of the cells during the reorientation events (Bubendorfer et al., 2014), an indirect effect on cell navigation is conceivable.

In addition to MotL, other PilZ domain-only proteins have been implicated in the regulation of the flagella-mediated motility (see Figure 1C). The single PilZ domain protein DgrA was shown to be a c-di-GMP-dependent negative regulator of the flagella-mediated motility in *C. crescentus* (Christen et al., 2007), although the overall homology to the PilZ domains of other flagellar regulators is rather small. The mechanism by which DgrA exerts this control remains obscure so far, but it is suggested to occur via the levels of the motor protein FliL, which we have shown to be not the case for MotL. In addition, also in *Sinorhizobium meliloti*, a PilZ domain-only protein, McrA, has been demonstrated to negatively affect swimming in dependence of c-di-GMP binding, however, also for McrA, the mechanism is still unsolved (Schäper et al., 2016). Thus, MotL/DgrA/McrA-like flagellar motor regulators may be common among bacteria but further studies are required to determine their exact underlying mechanism.

## Materials and Methods

### Bacterial Strains, Growth Conditions, and Media

Bacterial strains used in this study are summarized in Supplementary Table 1. *Shewanella putrefaciens* CN-32 (CN-32) strains were cultivated at 30°C in LB, and *E. coli* strains were cultured at 37°C in LB. When appropriate, media were supplemented with 50 μg ml^–1^ of kanamycin, 40 ng ml^–1^ of anhydrotetracyclin, 10% (w/v) sucrose, or 1.5% (w/v) agar. Soft agar plates were prepared with LB and 0.3% (w/v) agar, and when necessary, 50 μg ml^–1^ of kanamycin and 40 ng ml^–1^ of anhydrotetracyclin was added. *E. coli* WM3064 cultures were supplemented with 2,6-diamino-pimelic acid (DAP) to a final concentration of 300 μM.

### Strain and Vector Constructions

Bacterial strains and plasmids used in this study are listed in Supplementary Tables 1 2. Construction of Plasmids was performed using the method of Gibson assembly (Gibson et al., 2009). The corresponding oligonucleotides (Sigma-Aldrich, Taufkirchen, Germany) used for cloning are listed in Supplementary Table 3. All kits for preparation and purification of nucleic acids (VWR International GmbH, Darmstadt, Germany) and enzymes (Fermentas, St. Leon-Rot, Germany) were used according to the standard manufacturers’ protocols. In-frame deletions or chromosomal integration of fusions in *S. putrefaciens* CN-32 were generated using the suicide vector pNTPS-138-R6K as previously described (Lassak et al., 2010). Plasmids were transferred into *S. putrefaciens* cells via conjugation using *E. coli* WM3064 as a donor. To generate markerless in-frame deletions, 500–700 bp fragments flanking the target gene were amplified and fused to create a deletion leaving only eight codons of the 5′- and 3′-termini of the corresponding gene. To complement the deletion mutant, full-length *motL* was reinserted into its native chromosomal position. To generate a c-di-GMP-insensitive version of MotL, the highly conserved c-di-GMP-binding residues in the PilZ domain (R29A; R33A; D73A; S75A; and G78A) were substituted by alanine, and the resulting protein was named MotL_*NCB*_ (for *n*o *c*-di-GMP-*b*inding). For N-terminal fluorescent fusions the genes of *motL* (or *motL*_*NCB*_) and *mcherry* (or *sfgfp*) with a flexible GSGS linker-encoding sequence between these genes were re-integrated into the appropriate deletion strains. In order to overproduce a fluorescent version of MotL (or MotL_*NCB*_), the vector pBTOK (Rossmann et al., 2015) with the same GSGS linker between the protein and fluorescent tags was used.

### Fluorescence Microscopy

Strains were cultivated overnight in the LB medium and sub cultured the next day until they reached the exponential growth phase (OD_600_ = 0.2–0.3). Then, 5 μl of culture were spotted on an agarose pad [PBS buffer solified with 1% (w/v) agarose]. Fluorescence images were acquired using a Leica DMI 6000 B inverse microscope (Leica, Wetzlar, Germany) equipped with an SCMOS camera (Visitron Systems, Puchheim, Germany) and a HC PL APO 100×/1.4 oil PH3 objective. Image processing and analysis was carried out using the ImageJ-based Fiji tool (Schindelin et al., 2012).

### Hook Stain

Fluorescent staining of lateral hook structures (FlgE_2_–Cys) (Schuhmacher et al., 2015) were prepared as previously described (Guttenplan et al., 2013; Rossmann et al., 2015) using Alexa Fluor 488-maleimide (Molecular Probes, Life Technologies) or iFluor 568-maleimide (AAT Bioquest) for staining.

### Motility Assays

#### Determination of Swimming Speed

Cells of CN-32 strains from overnight cultures were sub inoculated in 10 ml of LB with an OD_600_ of 0.05. After reaching the exponential growth phase (OD_600_ = 0.2–0.3), a 100 μl aliquot was placed under a coverslip fixed by four droplets of silicone (baysilone, VWR International GmbH, Darmstadt, Germany) to generate a space of 1–2 mm width. Movies of 120 frames were taken at room temperature with a Leica DMI 6000 B inverse microscope (Leica, Wetzlar, Germany) equipped with an SCMOS camera (Visitron Systems, Puchheim, Germany) and a HCX PL APO 100×/1.4 objective. The speed of at least 500 cells per strain was quantified using the MTrackJ plugin of Fiji (Meijering et al., 2012). Significance was tested using ANOVA (*p* = 0.05) in R version 3.0.1.

#### Spreading on Soft-Agar Plates

Spreading ability in semi-solid environments was analyzed by placing 4 μl of an exponentially grown planktonic culture on soft agar plates [LB supplemented with 0.3% (w/v) agar and, if applicable, 50 μg ml^–1^ of kanamycin and 40 ng ml^–1^ of anhydrotetracyclin) followed by an incubation period of 24 h at room temperature. Strains that were to be directly compared were always placed on the same plate. Plates were scanned for documentation using an Epson V700 Photo Scanner.

### Western Blotting

Production and stability of proteins fused to a fluorophore were determined by immunoblot analysis. Protein lysates were prepared from exponentially growing cultures and uniformly adjusted to an OD_600_ of 10. Protein separation and immunoblot detection were carried out as previously described (Bubendorfer et al., 2012; Binnenkade et al., 2014) using polyclonal antibodies raised against the FLAG-tag (Sigma-Aldrich/Merck, Darmstadt, Germany). Signal detection was carried out using the SuperSignal^®^ West Pico Chemiluminescent Substrate (Thermo Scientific, Schwerte, Germany), and was documented using a FUSION-SL chemiluminescence imager (Peqlab, Erlangen, Germany).

### *In vitro* Interaction Studies on MotL and the Flagellar C-Ring

#### Protein Purification

The protein constructs of interest (with either His or GST tags) were overproduced in *E. coli* BL21 (DE3) competent cells. Cell cultures were grown in the LB medium at 30°C overnight, and shaken at 180 rpm. 1% (w/v) lactose monohydrate was used for induction. Cells were harvested, lysed by microfluidizer (M110-L, Microfluidics), and centrifuged to pellet cell debris. The supernatant was then loaded onto a GE Healthcare HisTrapFF affinity column (for His-tagged proteins) or a GSTrap affinity column (for GST-tagged proteins). For the His-tagged proteins, the lysis and wash buffer contained 20 mM HEPES pH 8.0, 250 mM NaCl, 20 mM KCl, 20 mM MgCl_2_, and 40 mM imidazole, while the imidazole concentration in the elution buffer was increased to 500 mM. For the GST-tagged proteins, the lysis and wash buffer contained 20 mM HEPES pH 7.5, 200 mM NaCl, 20 mM KCl, 20 mM MgCl_2_, and the elution buffer additionally contained 50 mM *Tris*-HCl pH 8.0 and 20 mM of L-glutathione. After elution, the protein was purified by size exclusion chromatography (SEC) using an S200 Sepharose column and GE Lifesciences ÄKTA Prime and Purifier systems. After purification, the proteins were concentrated using the Amicon Ultra-15 spin concentrators (10 kDa cutoff point) and flash-frozen to be used in *in vitro* GST pulldown assays.

#### *In vitro* GST Pulldown Assays

The assays were performed using spin columns from the company MobiTec. After assembly of the columns, 500 μL of GST-pulldown buffer (20 mM HEPES pH 7.5, 200 mM NaCl, 20 mM KCl, 20 mM MgCl_2_, and 0.6 μM Tween20) was added into each column, and then 30 μl of resuspended bead slurry was pipetted in (GST-Sepharose beads). The resulting suspension was centrifuged for 1 min at 4,000 rpm to wash the beads, which were then resuspended in 500 μL of the GST-pulldown buffer. The beads were exposed to 1 nmol of a GST-tagged protein, incubated with the protein on a rotation machine for 15 min and centrifuged under the same conditions as above. The interaction partner protein was then added (10 nmol), and incubation period on the rotation machine was carried out for 30 min. The sample was centrifuged again and then washed three times with 500 μl of the GST-pulldown buffer. The elution was carried out with 40 μl of the GSH elution buffer (containing 50 mM *Tris*-HCl pH 8.0 and 20 mM L-glutathione). The samples were then analyzed by the SDS-PAGE gel separation and staining.

### Bacterial Two-Hybrid Assays

To determine the potential interaction between MotL and proteins of the lateral flagellar motor, we used a bacterial two-hybrid (BACTH) system (Euromedex) essentially as previously described (Karimova et al., 1998, 2005). The proteins of interest were genetically fused N- and C-terminally to the T25 and T18 fragments of the adenylate cyclase catalytic domain from *B. pertussis*. If an *in vivo* interaction of the fusion proteins occurs, the two adenylate cyclase fragments T18 and T25 will come into a sufficiently close proximity to become active and produce cAMP. The according hybrid genes were assembled in the expression plasmids pKT25, pKNT25, pUT18, and pUT18C. Appropriate plasmid pairs were co-transformed in *E. coli* BTH101, which lacks adenylate cyclase activity. The cells were grown and plated on LB_*AmpKm*_ plates containing isopropyl β-*D*-1-thiogalactopyranoside (IPTG; 0.5 mM) for induction of protein production and 5-bromo-4-chloro-3-indolyl β-*D*-galactopyranoside (X-Gal; 40 μg ml^–1^) and for simple detection of adenylate cyclase activity. The plates were incubated for 48 h at 30°C, and protein interaction was scored by the blue coloration of the corresponding colonies. The empty plasmids were used as a negative control while the plasmid pair pKT25-ZIP and pUT18C-ZIP served as a positive control, and both were present on any assay plate.

### c-di-GMP Binding Assays

#### Enrichment of MotL Variants by Magnetic Beads Pulldown

GFP-tagged variants of MotL were directly isolated from *S. putrefaciens* CN-32 cell lysate through protein-protein interaction with GFP-Trap_MA beads (ChromoTek GmbH, Planegg, Germany). Cells were cultivated overnight in the LB medium containing 50 μg ml^–1^ kanamycin and sub cultured and induced with 40 ng ml^–1^ anhydrotetracyclin the next day. The entire culture was harvested after reaching an OD_600_ = 1 and the immunoprecipitation was carried out according to the manufacturer’s protocol.

#### Production of Radioactively Labeled c-di-GMP

Radiolabeled c-di-GMP was synthesized from [α^32^-P]-GTP (Hartmann Analytik, Germany) by the mutated diguanylatecyclase WspR (R242A) as previously described (Sambanthamoorthy et al., 2012; Srivastava and Waters, 2015). For the heterologous production of WspR (R242A), *E. coli* BL21 (DE3) (New England Biolabs, Frankfurt, Germany) cells harboring the applicable pET24C overexpression plasmid were grown at 37°C in the LB medium containing 50 μg ml^–1^ kanamycin to an OD_600_ = 0.3. Then, the cells were induced with D-(+)-lactose-monohydrate (12.5 g⋅l^–1^) and incubated overnight at 15°C. The entire culture was harvested and the cell pellet was resuspended in buffer A [50 mM *Tris*-HCl, 250 mM NaCl, 25 mM KCl, 5 mM MgCl2, 0.5 mM dithiothreitol (DTT), 0.01% sodium azide, 5% glycerol, and 40 mM imidazole] and lysed by sonification (Bandelin Sonoplus). After centrifugation (20,000 rpm, 30 min, 4°C), the supernatant was filtered and loaded on a 5-ml HisTrap column (GE Healthcare), equilibrated with five column volumes (CV) of buffer A. The column was washed with 5 CV of buffer A before the protein was eluted with 5 CV of buffer B (buffer A with an imidazole concentration of 300 mM). Protein concentration was determined by spectrophotometry (NanoDrop Lite, Thermo Scientific) and the proteins were used immediately or stored for a short time at 4°C.

#### Binding Assays

For binding assays, 1.5 μM of the appropriate protein was incubated together with 2 μl of radiolabeled c-di-GMP or GTP in a binding buffer (5 mM *Tris-*HCl, 12.5 mM NaCl, 2.5 mM MgCl_2_; and final volume of 50 μl) for 5 min at RT before 10 μl were spotted on a nitrocellulose membrane (in triplicates). After drying, the membrane was washed three times with the binding buffer, tried, and exposed on phosphorimaging screens (Bio-Rad) and analyzed with the 1D-Quantity One software (Bio-Rad).

## Data Availability Statement

The original contributions presented in the study are included in the article/Supplementary Material, further inquiries can be directed to the corresponding author.

## Author Contributions

KT and GB conceptualized the study and provided resources and funding. AP, MS, VB, TR, PK, and TL conducted the experiments. AP, MS, VB, and KT wrote the manuscript. All authors read and commented on the manuscript.

## Conflict of Interest

The authors declare that the research was conducted in the absence of any commercial or financial relationships that could be construed as a potential conflict of interest.
