# Corrigendum: The Stand-Alone PilZ-Domain Protein MotL Specifically Regulates the Activity of the Secondary Lateral Flagellar System in *Shewanella putrefaciens*

**DOI:** 10.3389/fmicb.2021.726192

**Published:** 2021-07-16

**Authors:** Anna Pecina, Meike Schwan, Vitan Blagotinsek, Tim Rick, Patrick Klüber, Tabea Leonhard, Gert Bange, Kai M. Thormann

**Affiliations:** ^1^Department of Microbiology and Molecular Biology, Justus-Liebig-Universität Gießen, Giessen, Germany; ^2^Department of Chemistry, SYNMIKRO Research Center, Philipps-University Marburg, Marburg, Germany

**Keywords:** flagella, c-di-GMP, flagellar motor, YcgR, *Shewanella*, PilZ domain, lateral flagella

In the original article, there was a mistake in Figure 1 as published. **The alignment shown in 1D for DgrA was not correct. We also updated the signature motif for the c-di-GMP-bindig motif of PilZ domains with a more recent one**. The corrected Figure 1 appears below.

**Figure 1:**
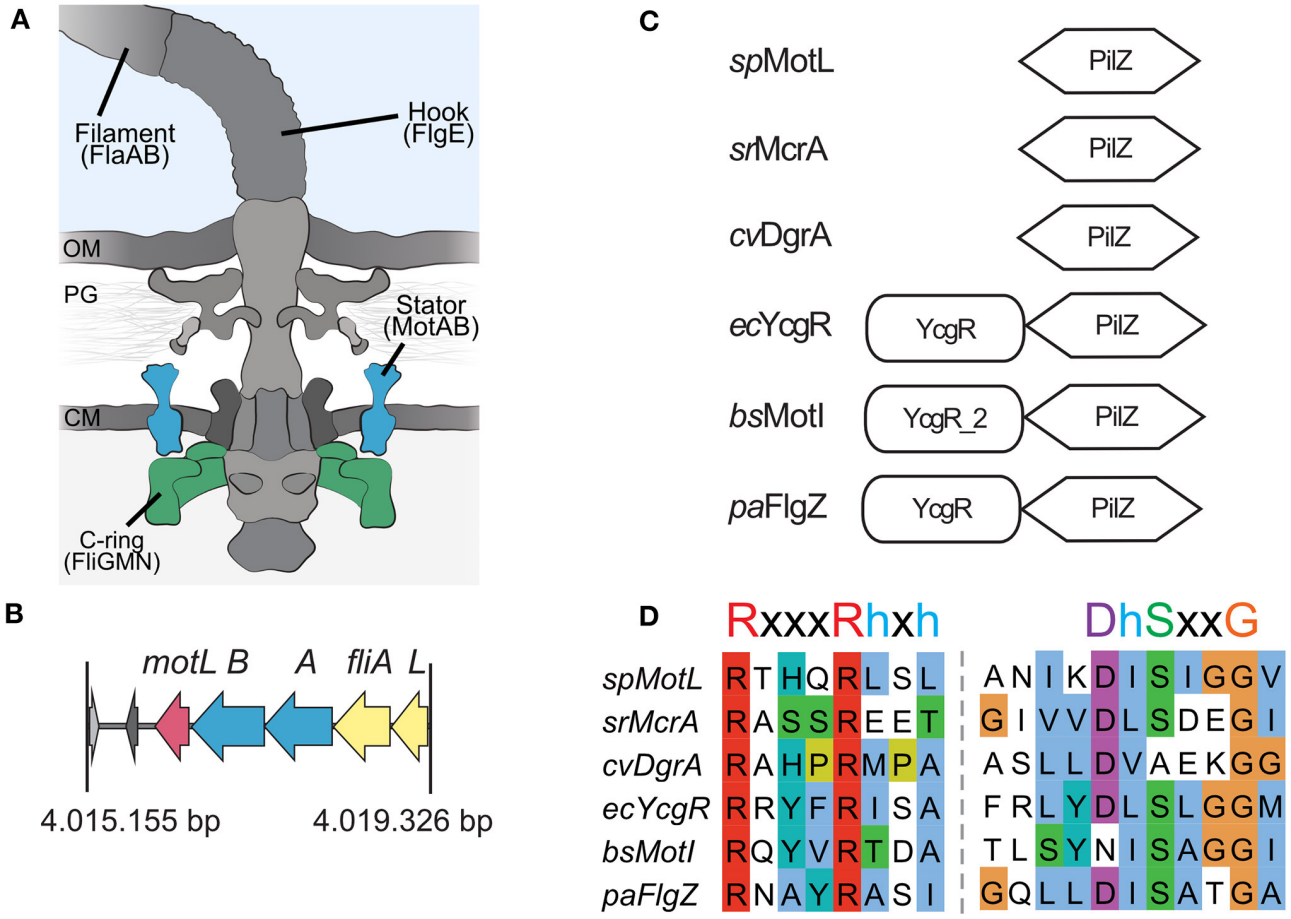


In the original article, there was an error. **In the text appeared an older version of the c-di-GMP-binding motif within PilZ domains. We have updated the information along with the corresponding reference**.

A correction has been made to ***Results***, ***Identification of a flagellar motor effector*** ***protein in Shewanella putrefaceins***, ***2nd paragraph***:

Sputcn32_3446, annotated as a PilZ domain, is located 34 bp downstream of *motB* and transcribed in the same direction (Figure 1B). The gene is 435 bp in length and encodes a protein of 144 aa with an estimated molecular mass of 16.6 kDa and a theoretical pI of 5.94. The protein is thus much smaller than YcgR (244 aa), FlgZ (263), and MotI (217aa) as an N-terminal YcgR domain is not present (Figure 1C). The predicted c-di-GMP-binding motifs (**RxxxRhxh, DhSxxG; Galperin and Chou**, **2020**) are fully conserved (Figure 1D). The protein is conserved in a number *Shewanella* species that possess dual flagellar systems, and the gene it is always located downstream of *motB*. Potential homologs of Sputcn32_3446 are also present in some species of *Aeromonas* and *Vibrio*, but absent from the well-characterized *V*. *parahaemolyticus* and *V. alginolyticus*, which also possess two distinct flagellar systems. We henceforth referred to the protein as MotL, relating to its location within the lateral flagellar gene operon and its differences to YcgR, FlgZ, and MotI with respect to the protein sequence and absence of further domains.

The authors apologize for this error and state that this does not change the scientific conclusions of the article in any way. The original article has been updated.
