# Supplementary material for: The Stand-Alone PilZ-Domain Protein MotL Specifically Regulates the Activity of the Secondary Lateral Flagellar System in Shewanella putrefaciens
Source: Front Microbiol. 2021 Jun 1;12:668892. doi: 10.3389/fmicb.2021.668892 (PMC8203827; doi:10.3389/fmicb.2021.668892)
Supplement: Supplementary file 1 [file Data_Sheet_1.pdf]

Supplementary Material

Table S1: Strains used in this study

strain	relevant genotype or phenotype	source or reference
Escherichia coli		
DH5α λpir	Φ80dlacZΔM15 Δ(lacZYA-argF) U196 recA1 hsdR17 deoR thi-1 supE44 gyrA96 relA1/λpir	(Miller and Mekalanos, 1988)
WM3064	hrB1004 pro thi rpsL hsdS lacZΔM15 RP4–1360Δ(araBAD)567 ΔdapA1341::[erm pir(wt)]	W. Metcalf, University of Illinois
BTH101	F-, cya-99, araD139, galE15, galK16, rpsL1 (Strr), hsdR2, mcrA1, mcrB1	(Karimova et al., 2002)
BL21star (DE3)	fhuA2 [lon] ompT gal (λ DE3) [dcm] ΔhsdS λ DE3 = λ sBamHIo ΔEcoRI-B int::(lacI::PlacUV5::T7 gene1) i21 Δnin5	Euromedex, Frankreich InvitrogenTM, Thermo Fischer Scientific
***Shewanella putrefaciens* CN-32**		
WT	wild type	(Fredrickson et al., 1998)
ΔP	deletion of polar flagellins *flaAB*$_1$(ΔSputcn32_2585-ΔSputcn32_2586)	(Bubendorfer et al., 2014)
ΔL	deletion of lateral flagellins *flaAB*$_2$ (ΔSputcn32_3455-ΔSputcn32_3456)	(Bubendorfer et al., 2014)
ΔP ΔL	deletion of polar and lateral flagellins	Kühn et al 2018
Δ*motL*	deletion of the lateral flagellar brake (ΔSputcn32_3446)	this study
Δ P Δ*motL*	deletion of polar flagellins and the lateral flagellar brake	this study
Δ P Δ*motL* KI *gfp-motL*	chromosomal insertion of N-terminal sfGfp-tagged MotL (lateral flagellar brake) and deletion of polar flagellins	this study
ΔLΔ*motL*	deletion of polar flagellins and the lateral flagellar brake	this study
Δ P ΔL Δ*motL*	deletion of polar and lateral flagellins and the lateral flagellar brake	this study

Δ*fliM*$_2$ KI *fliM*$_2$-mCherry	chromosomal insertion of C-terminal mCherry-tagged FliM$_2$ (rotor protein)	(Bubendorfer et al., 2012)
Δ*fliM*$_2$ KI *fliM*$_2$-mCherry Δ*motL* KI gfp-*motL*	chromosomal insertion of C-terminal mCherry-tagged FliM$_2$ (rotor protein) and chromosomal insertion of N-terminal sfGfp-tagged MotL (lateral flagellar brake)	this study
Δ*motB* KI mCherry-*motB*	chromosomal insertion of N-terminal mCherry-tagged MotB (stator protein)	(Bubendorfer et al., 2012)
Δ*motB* KI mCherry-*motB* Δ*motL* KI gfp-*motL*	chromosomal insertion of N-terminal mCherry-tagged MotB (stator protein) and chromosomal insertion of N-terminal sfGfp-tagged MotL (lateral flagellar brake)	this study
flgE$_2^{T242C}$	chromosomal insertion of cysteine-labeled lateral hook protein FlgE$_2$	(Schuhmacher et al., 2015)
fliL$_2$*-FLAG*	chromosomal insertion of C-terminal FLAG-tagged FliL$_2$	this study
Δ*motL fliL*$_2$-FLAG	chromosomal insertion of C-terminal FLAG-tagged FliL$_2$ and deletion of the lateral flagellar brake	this study

Table S2: Plasmids used in this study

Plasmid	Genotype/ insert/ purpose	Source of reference
pNPTS138-R6KT	*mob*RP4+, *ori*-R6K, *sacB*, beta-galactosidase fragment alpha, Km^r; suicide plasmid for in-frame deletions or integrations	(Lassak et al., 2010)
pBTOK	pBBR1-MCS2 backbone (pBBR origin, Km^r); TetR, Promoter and multiple cloning site of pASK-IBA3plus and *E.coli* rrnB1 T1 and lambda phage T0 terminator; overproduction plasmid inducible with anhydrotetracycline (AHT)	(Rossmann et al., 2015)
pET21-*sfGfp*	template for super folder green fluorescend protein (sfGFP)	(Pédelacq et al., 2006)
put18	Ori-ColE1, Amp^r, C-terminal fusion of the T18 fragment to the protein of interest	Euromedex, (Karimova et al., 1998)
pUT18c	Ori-ColE1, Amp^r, N-terminal fusion of the T18 fragment to the protein of interest	Euromedex, (Karimova et al., 1998)
pKT25	Ori-p15a, Km^r, ,N-terminal fusion of the T25 fragment to the protein of interest	Euromedex, (Karimova et al., 1998)
pKNT25	Ori-p15a, Km^r, C-terminal fusion of the T25 fragment to the protein of interest	Euromedex, (Karimova et al., 1998)
pET24c	overproduction plasmid inducible with lactose, Km^r	EMD Biosciences
pET24d	overproduction plasmid inducible with lactose, Km^r	EMD Biosciences
pET16b	overproduction plasmid inducible with lactose, Amp^r	EMD Biosciences
pGAT3	overproduction plasmid (GST) inducible with lactose	(Peränen et al., 1996)
***in frame* deletion constructs**		
pNPTS *motL* KO	*motL* (Sputcn32_3446) deletion fragment in pNPTS138-R6KT, Km^r	this study
**Insertion constructs**		
pNPTS KI gfp-*motL*	insertion fragment of *sfgfp-motL* (Sputcn32_3446) in pNPTS138-R6KT, Km^r, N-terminal fusion	this study
pNPTS *fliL*$_2$-FLAG	insertion fragment of *fliL*$_2$-FLAG in pNPTS138-R6KT, Km^r, C-terminal fusion	this study

**Overproduction constructs**		
pBTOK sfGFP-*motL*	overproduction plasmid for sfGFP-MotL (Sputcn32_3446), N-terminal sfGFP fusion, inducible with AHT, Km^r	this study
pBTOK sfGFP-$motL_{NCB}$	overproduction plasmid for sfGFP-$MotL_{NCB}$ (Sputcn32_3446), N-terminal sfGFP fusion, inducible with AHT, Km^r	this study
pBTOK *pdeH*	overproduction plasmid for the phosphodiesterase PdeH of *E. coli*, inducible with AHT, Km^r	this study
pBTOK *dgcA*	overproduction plasmid for the diguanylatecyclase DgcA of *V. cholerae*, inducible with AHT, Km^r	this study
pBTOK sfGFP-*motL* -*pdeH*	overproduction plasmid for sfGFP-MotL in concert with PdeH, inducible with AHT, Km^r	this study
pBTOK sfGFP-*motL*-*dgcA*	overproduction plasmid for sfGFP-MotL in concert with DgcA, inducible with AHT, Km^r	this study
pBTOK sfGFP-$motL_{NCB}$-*pdeH*	overproduction plasmid for sfGFP-$MotL_{NCB}$ in concert with PdeH, inducible with AHT, Km^r	this study
pBTOK sfGFP-$motL_{NCB}$ -*dgcA*	overproduction plasmid for sfGFP-$MotL_{NCB}$ in concert with DgcA, inducible with AHT, Km^r	this study
pet24c $wspR^{R242A}$	overproduction plasmid for the diguanylatecyclase $WspR^{R242A}$ of *P. aeruginosa*, inducible with lactose, Km^r	this study
pet24c 6xHis-*motL*	overproduction plasmid for the Histidin-tagged version of MotL, inducible with lactose, Km^r	this study
pet24c 6xHis-$motL_{NCB}$	overproduction plasmid for the Histidin-tagged version of $MotL_{NCB}$, inducible with lactose, Km^r	this study
**BACTH constructs**		
pUT18 *motL*	C-terminal fusion of the T18 fragment to MotL (Sputcn32_3446), Amp^r	this study
pUT18C *motL*	N-terminal fusion of the T18 fragment to MotL, Amp^r	this study
pKT25 *motL*	N-terminal fusion of the T25 fragment to MotL, Km^r	this study
pKNT25 *motL*	C-terminal fusion of the T25 fragment to MotL, Km^r	this study
pUT18 $motL_{NCB}$	C-terminal fusion of the T18 fragment to $MotL_{NCB}$ (Sputcn32_3446), Amp^r	this study
pUT18C $motL_{NCB}$	N-terminal fusion of the T18 fragment to $MotL_{NCB}$, Amp^r	this study

pKT25 *motL*$_{NCB}$	N-terminal fusion of the T25 fragment to MotL$_{NCB}$, Km^r	this study
pKNT25 *motL*$_{NCB}$	C-terminal fusion of the T25 fragment to MotL$_{NCB}$, Km^r	this study
pUT18 *fliM*$_2$	C-terminal fusion of the T18 fragment to FliM$_2$ (Sputcn32_3479), Amp^r	this study
pUT18 *fliM*$_2$	N-terminal fusion of the T18 fragment to FliM$_2$, Amp^r	this study
pKT25 *fliM*$_2$	N-terminal fusion of the T25 fragment to FliM$_2$, Km^r	this study
pKNT25 *fliM*$_2$	C-terminal fusion of the T25 fragment to FliM$_2$, Km^r	this study
pUT18 *fliN*$_2$	C-terminal fusion of the T18 fragment to FliN$_2$ (Sputcn32_3480), Amp^r	this study
pUT18C *fliN*$_2$	N-terminal fusion of the T18 fragment to FliN$_2$, Amp^r	this study
pKT25 *fliN*$_2$	N-terminal fusion of the T25 fragment to FliN$_2$, Km^r	this study
pKNT25 *fliN*$_2$	C-terminal fusion of the T25 fragment to FliN$_2$, Km^r	this study
pUT18 *fliG*$_2$	C-terminal fusion of the T18 fragment to FliG$_2$ (Sputcn32_3475), Amp^r	this study
pUT18C *fliG*$_2$	C-terminal fusion of the T18 fragment to FliG$_2$, Amp^r	this study
pKT25 *fliG*$_2$	N-terminal fusion of the T25 fragment to FliG$_2$, Km^r	this study
pKNT25 *fliG*$_2$	C-terminal fusion of the T25 fragment to FliG$_2$, Km^r	this study
pUT18 *motB*	C-terminal fusion of the T18 fragment to MotB (Sputcn32_3447), Amp^r	this study
pUT18C *motB*	N-terminal fusion of the T25 fragment to MotB, Amp^r	this study
pKT25 *motB*	N-terminal fusion of the T25 fragment to MotB, Km^r	this study
pKNT25 *motB*	C-terminal fusion of the T25 fragment to MotB, Km^r	this study
pUT18 *motA*	C-terminal fusion of the T18 fragment to MotA (Sputcn32_3448), Amp^r	this study
pUT18C *motA*	N-terminal fusion of the T25 fragment to MotA, Amp^r	this study
pKT25 *motA*	N-terminal fusion of the T25 fragment to MotA, Km^r	this study
pKNT25 *motA*	C-terminal fusion of the T25 fragment to MotA, Km^r	this study

Table S3: Oligonucleotides used in this study

Oligonucleotide	Sequence (5´-3´)
**deletion of *motL***	
NheI_Sputcn32_3446_fw	GTA GCT AGC CAG GGT ATC CGT ATT TTG ATC C
OL_Sputcn32_3446_KO_rv	TGC CCT ATA TCT CTT CAT ACA TAT TCA TAG TCA TAC C
OL_Sputcn32_3446_KO _fw	GTA TGA AGA GAT ATA GGG CAT GCA ATG GCT GC
PspOMI_Sputcn32_3446_rv	TCC GGG CCC GTA TCA ACC GTG GTA CTC TGC
Check_3446_KO_fw	TGG TGC TAA GCG AAG TAG AAG C
Check_3446_KO_rev	CTG TTT TAG CAA GGC AAT TGA ATC G
**deletion of *fliG$_2$***	
EcoRV_FliG_up_fw	CAA GCT TCT CTG CAG GAT GCG GAT TTT ATC CTC AGC CAG
OL_FliG_up_rv	GGA TAA TTA CGTC GTT GTC TAA TGG AAA ACA AGC
OL_FliG_down_fw	AGA CAA CGA CGTA ATT ATC CAT CTT AAT TTC GAT TGA G
EcoRV_FliG_down_rv	GAA TTC GTG GAT CCA GAT GTC TCA GTA CTC ATC AAT AGC G
Check_fliG2 KO_fw	ATC TGC AAT CAG ATT CCA GCC G
Check_fliG2 KO_rev	GAA CCA GGA TCA CCT TTA ACG G
**insertion of sfGFP-*motL***	
EcoRV_3446_up_fw	CAA GCT TCT CTG CAG GAT GGC GGA GAA TAT CAC TGT CAC
OL_3446_up_rv	CTT TGC TCA T AGT CAT ACC TAA CAA ATG AGT ACA AG
OL_up_sfGFP_fw	AGG TAT GAC T ATG AGC AAA GGA GAA GAA CTT TTC
OL_down_3446_rv	ATT GCA TGC C TTA TAT TTT GGC TCG TAA TTT AAT TGC G
OL_3446_down_fw	CAA AAT ATA A GGC ATG CAA TGG CTG CAT CTT
EcoRV_3446_down_rv	GAA TTC GTG GAT CCA GAT CAT CAT GCA ACA CAC TCG TGG
Check_3446_KO_fw	TGG TGC TAA GCG AAG TAG AAG C
Check_3446_KO_rev	CTG TTT TAG CAA GGC AAT TGA ATC G
**insertion of *fliL$_2$*-FLAG**	
EcoRV-FliL2 FLAG-fwd	GCG AAT TCG TGG ATC CAG ATA AAA TGG CGC CGA TGT GAT GAC
OL-FliL2 FLAG-rev	AAT ATC ATG ATC TTT ATA ATC GCC ATC ATG ATC TTT ATA ATC CTG GAT CAC CAT ACG GGT AAA AAG
OL-FliL2 FLAG-fwd	ATT ATA AAG ATC ATG ATA TTG ATT ATA AAG ATG ATG ATG ATA AAT AAG GGG CCG ATA TGA CGA CAG
EcoRV-FliL2 FLAG-rev	GCC AAG CTT CTC TGC AGG ATA CAT CAT TGC CTC TAT CGA CCG
Check-FliL2 FLAG-fwd	TTT CAG CAG GCT GCC ATA ATG C
Check_Flag C-term	GAT CAT GAT GGC GAT TAT AAA GAT C
**overproduction of sfGFP-*motL***	
XbaI-nC_sf_GFP_fw	AAT GAA TAG TTC GAC AAA AAT AGG AGG CTT AGT CCA T ATG AGC AAA GGA GAA GAA CTT TTC ACT G
OL__sfGFP_Sputcn32_3446_rv	CAT ACA TAT T CGA GCC GGA TCC TTT GTA GAG CTC ATC CAT CAT C
OL_sfGFP_Sputcn32_3446_fw	CGG CTC GAA TAT GTA TGA AGA GTT TGT ACA TTC
PspOMI-nC_3446_rev	GGA GTC CAA GCT CAG CTA ATG TTA TAT TTT GGC TCG TAA TTT AAT TGC GTC
**overproduction of sfGFP-*motL$_{NCB}$***	
XbaI-nC_sf_GFP_fw	AAT GAA TAG TTC GAC AAA AAT AGG AGG CTT AGT CCA T ATG AGC AAA GGA GAA GAA CTT TTC ACT G
OL__sfGFP_Sputcn32_3446_rv	CAT ACA TAT T CGA GCC GGA TCC TTT GTA GAG CTC ATC C
OL_sfGFP_Sputcn32_3446_fw	ATC CGG CTC G AAT ATG TAT GAA GAG TTT GTA CAT TC

1. Binding site rv	CGT TGA TAA ATT AAC CGT TAA GCC ATC ACA GGC AAA CGC TAA TGG CAC TCT TTT ATC ATG TCG TAG GCT GAG TGC CTG ATG AGT TGC CCG ATT
2. Binding site fw	TTA ACG GTT AAT TTA TCA ACG ACT CGT TGG TTT ATC CTC ACG CCA CTC GGT ACC GCG AAC ATA AAA GCA ATT GCA ATT GGC
PspOMI_Sputcn32_3446_rev	GGA GTC CAA GCT CAG CTA ATG TTA TAT TTT GGC TCG TAA TTT AAT TGC GTC
**overproduction of sfGFP-*motL/*sfGFP-*motL*$_{NCB}$*_pdeH***	
XbaI-nC_sf_GFP_fw	AAT GAA TAG TTC GAC AAA AAT AGG AGG CTT AGT CCA T ATG AGC AAA GGA GAA GAA CTT TTC ACT G
3446_rv_OL_RBS2	TAA GCC TCC TTG CTA GCC TTA TAT TTT GGC TCG TAA TTT AAT TGC GTC
OL_3446_PdeH_fw	AAG GCT AGC A AGG AGG CTT AGT CCA T ATG ATA AGG CAG GTT ATC CAG CG
PspOMI_PdeH_rev	GGA GTC CAA GCT CAG CTA ATG TTA TAG CGC CAG AAC CGC CG
**overproduction of sfGFP-*motL/*sfGFP-*motL*$_{NCB}$*_dgcA***	
XbaI-nC_sf_GFP_fw	AAT GAA TAG TTC GAC AAA AAT AGG AGG CTT AGT CCA T ATG AGC AAA GGA GAA GAA CTT TTC ACT G
3446_rv_OL_RBS2	TAA GCC TCC T TG CTA GCC TTA TAT TTT GGC TCG TAA TTT AAT TGC GTC
OL_3446_VdcA_fw	AAG GCT AGC A AGG AGG CTT AGT CCA T GTG ATG ACA ACT GAA GAT TTC AAA AAA TC
PspOMI_VdcA_rev	GGA GTC CAA GCT CAG CTA ATG TTA GAG CGG CAT GAC TCG ATT G
**overproduction of *wspR*^{R242R}**	
NdeI his WspR fw	TTA ACT TTA AGA AGG AGA TAT ACA ATG CAC AAC CCT CAT GAG AGC AAG ACC
OL R242A rv	CCG AGG AGC CAC TGC AGC CCT CGC CGA TGG
OL R242A fw	CCA TCG GCG AGG GCT GCA GTG GCT CCT CGG
XhoI WspR rv	GTG GTG GTG GTG GTG GTG C TCA ATG GTG ATG GTG ATG GTG GCC CGC CGG GGC TGG CGG CAC
**overproduction of Histidin tagged motL/motL$_{NCB}$**	
NdeI FlgZ fw	TTA ACT TTA AGA AGG AGA TAT ACA ATG AAT ATG TAT GAA GAG TTT GTA CAT TCT
XhoI FlgZ his rv	GTG GTG GTG GTG GTG GTG C TCA ATG GTG ATG GTG ATG GTG TAT TTT GGC TCG TAA TTT AAT TGC GTC
**BACTH MotL**	
MotL fwPrimer	CTG CAG GTC GAC TCT AGA GAT GAA TAT GTA TGA AGA GTT TGT ACA TTC T
MotL rvPrimer	GAG CTC GGT ACC CGG GGT ATT TTG GCT CGT AAT TTA ATT GCG TC
MotL fwPrimerpKT	CAG GGT CGA CTC TAG AGA TGA ATA TGT ATG AAG AGT TTG TAC ATT CT
MotL rvPrimerpKT	TTA GTT ACT TAG GTA CCC GGG GTA TTT TGG CTC GTA ATT TAA TTG CGT C

**BACTH FliG$_2$**	
LZ062 B2H_3475_fw	CTG CAG GTC GAC TCT AGA GAT GGA TAA TTA CGC CCA AGC AGC
LZ063 B2H_3475_rv	GAG CTC GGT ACC CGG GGG ACA ACG ACC TGC TCT TCA AAT A
LZ064 B2H_3475_fwpKT	CAG GGT CGA CTC TAG AGA TGG ATA ATT ACG CCC AAG CAG C
LZ065 B2H_3475_rvpKT	TTA GTT ACT TAG GTA CCC GGG GGA CAA CGA CCT GCT CTT CAA ATA
**BACTH FliM$_2$**	
LZ066 B2H_3479_fw	CTG CAG GTC GAC TCT AGA GAT GAA GAT AAC CGC AAA AGC TCG
LZ067 B2H_3479_rv	GAG CTC GGT ACC CGG GGG CCA ATG TCG TTC TCC TCA TAC
LZ068 B2H_3479_fwpKT	CAG GGT CGA CTC TAG AGA TGA AGA TAA CCG CAA AAG CTC G
LZ069 B2H_3479_rvpKT	TTA GTT ACT TAG GTA CCC GGG GGC CAA TGT CGT TCT CCT CAT AC
**BACTH FliN$_2$**	
LZ070 B2H_3480_fw	CTG CAG GTC GAC TCT AGA GAT GAG GAG AAC GAC ATT GGC TG
LZ071 B2H_3480_rv	GAG CTC GGT ACC CGG GGT TCG TTA ATT GTC CCA TCC AGC G
LZ072 B2H_3480_fwpKT	CAG GGT CGA CTC TAG AGA TGA GGA GAA CGA CAT TGG CTG
LZ073 B2H_3480_rvpKT	TTA GTT ACT TAG GTA CCC GGG GTT CGT TAA TTG TCC CAT CCA GCG
**BACTH MotA**	
LZ054 B2H_3448_fw	C TGC AGG TCG ACT CTA GAG ATG AGT AAA CTA GTT GGG CTG TTA ATT
LZ055 B2H_3448_rv	GA GCT CGG TAC CCG GGG GCT TCT CTG CCC CTC CAT C
LZ056 B2H_3448_fwpKT	CA GGG TCG ACT CTA GAG ATG AGT AAA CTA GTT GGG CTG TTA ATT
LZ057 B2H_3448_rvpKT	T TAG TTA CTT AGG TAC CCG GGG GCT TCT CTG CCC CTC CATC
**BACTH MotB**	
LZ058 B2H_3447_fw	CTG CAG GTC GAC TCT AGA GAT GCT CCA TAA AAA TGA GCC GAT TAT T
LZ059 B2H_3447_rv	GAG CTC GGT ACC CGG GGA TGC GCG ATA GTC TGT CGT TTT ATA
LZ060 B2H_3447_fwpKT	CAG GGT CGA CTC TAG AGA TGC TCC ATA AAA ATG AGC CGA TTA TT
LZ061 B2H_3447_rvpKT	TTA GTT ACT TAG GTA CCC GGG GAT GCG CGA TAG TCT GTC GTT TTA TA
**BACTH check**	
pKT25-for	CAC TGA CGG CGG ATA TCG ACA TGT T
pKT25-rev	CCG CCG GAC ATC AGC GCC ATT C
pUT18-for	CCA GGC TTT ACA CTT TAT GCT TCC
pUT18-rev	GAC GCG CCT CGG TGC CCA CTG C
pKNT25-for	CCC AGG CTT TAC ACT TTA TGC TTC C
pKNT25-rev	GTT TTT TTC CTT CGC CAC GGC CTT G
pUT18C-for	CGG CGT GCC GAG CGG ACG TTC G
pUT18C-rev	TCA GCG GGT GTT GGC GGG TGT C

**Pulldown**	
DM318 (FliG2)	TTA CCA TGG GCC ACC ATC ACC ATC ACC ATA TGG ATA ATT ACG CCC AA GCA GC
DM319 (FliG2)	TTA ACT CGA GTT AGA CAA CGA CCT GCT CTT C
DM337 (FliM2)	TTA ACC ATG GGC AAG ATA ACC GCA AAA GCT CG
DM338 (FliM2)	TTA ACT CGA GTT AAT GGT GAT GGT GAT GGT GGC CAA TGT CGT TCT CCT C
DM339 (FliN2)	TTA ACC ATG GGC AGG AGA ACG ACA TTG GCT G
DM340 (FliN2)	TTA ACT CGA GTT ATT CGT TAA TTG TCC CAT CC

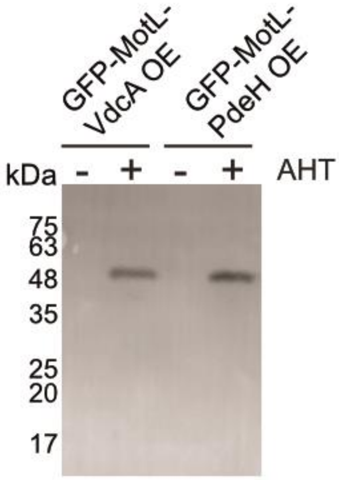

Supplementary Figure 1: sfGFP-tagged MotL is stably produced. Shown is a Western blot after PAGE separation of a crude extract of cells in which overproduction (OE) the fused MotL in concert with the diguanylate cyclase DgcA (left two lanes) or the phosphodiesterase PdeH (right lanes) is not induced (-) or induced (+). The protein was identified using an antibody directed against GFP.

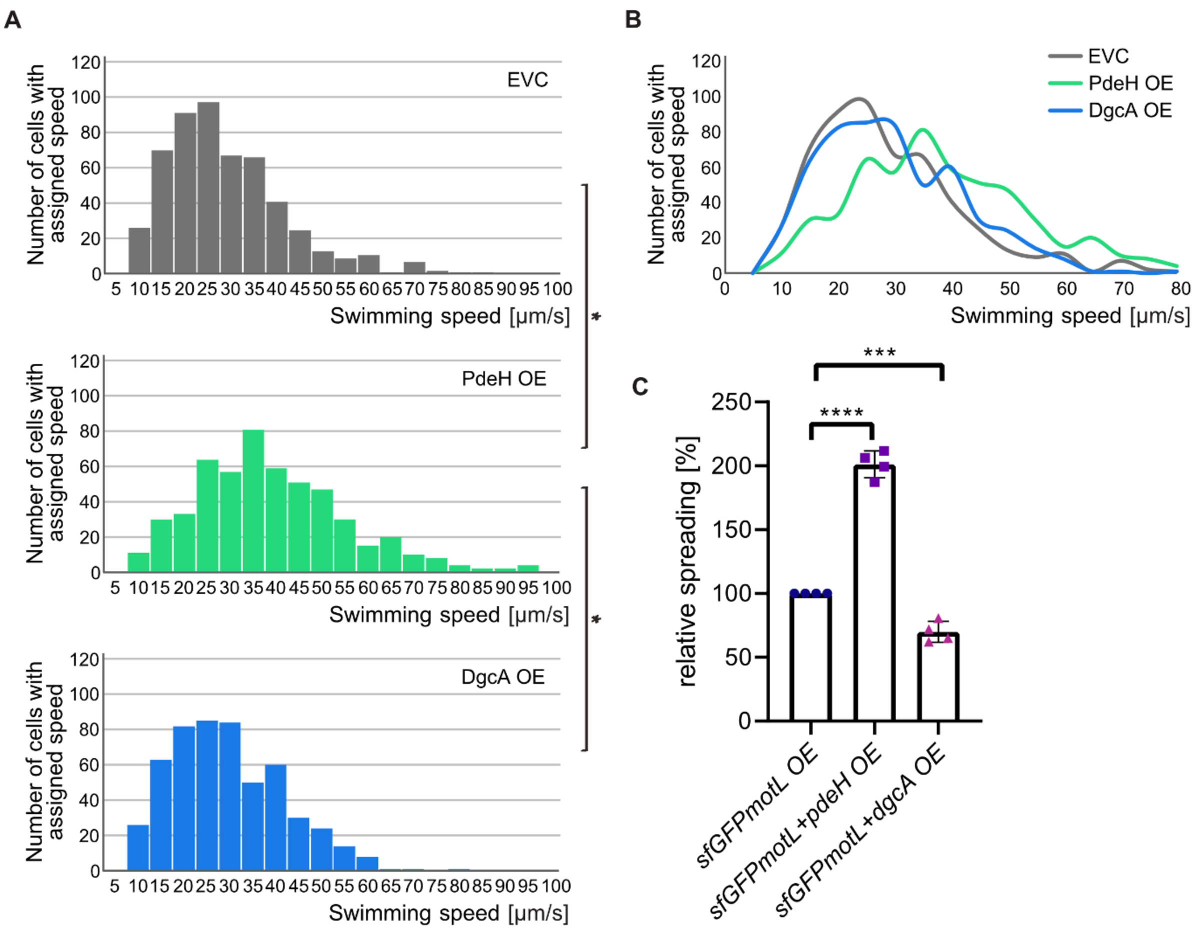

Supplementary Figure 2: MotL affects swimming and spreading mediated by lateral flagella in a c-di-GMP-dependent fashion. A) Swimming speed of *S. putrefaciens* cells lacking polar flagella (ΔL) overexpressing (OE) *gfp*, *gfp-motL* or *gfp-motLNCB*. B) Swimming speed of *S. putrefaciens* cells lacking polar flagella (ΔL) overexpressing (OE) *gfp-motL* in concert with *pdeH* or *dgcA*. N = 591. This figure is an alternative depiction of the data shown in Fig.4 of the main part. Shown is the number of cells that swim at the indicated speed. C) Relative spreading of the same mutant cells producing sfGFP-MotL together with PdeH or DgcA through soft agar. The asterisks indicate significant speed differences according to ANOVA.

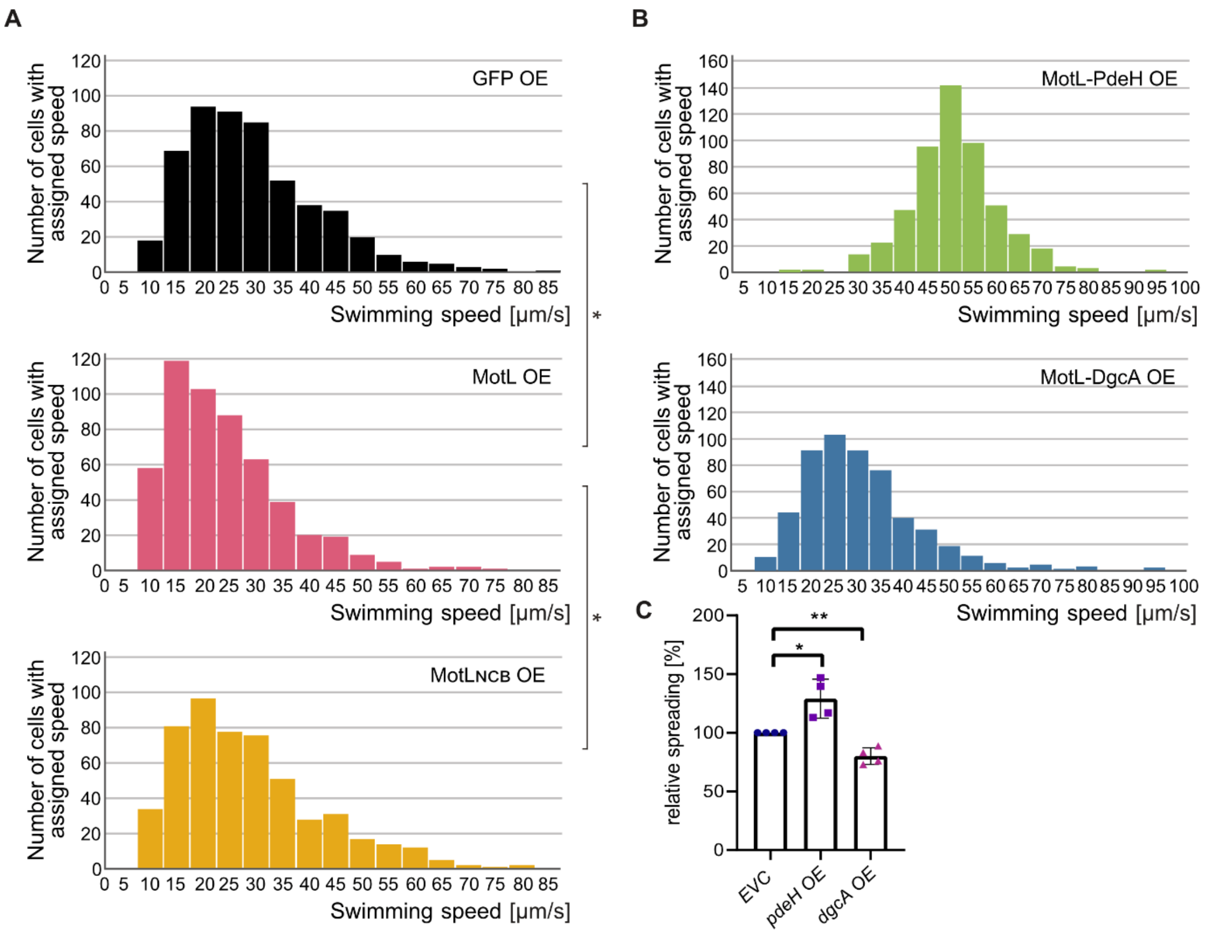

Supplementary Figure 3: Effect of *pdeH* or *dgcA* overexpression on the swimming speed and spreading of cells lacking polar flagella (ΔL) and *motL*. A) Effect of *pdeH* and *dgcA* overexpression on the swimming speed of *ΔLΔmotL* mutants. Shown is a depiction of the individual distributions. B) Data from A merged in a single graph. N = 591. EVC, empty vector control. C) Effect of *pdeH* and *dgcA* overexpression on spreading of *ΔLΔmotL* mutants through soft agar. The asterisks indicate significant differences in cell speed according to ANOVA.

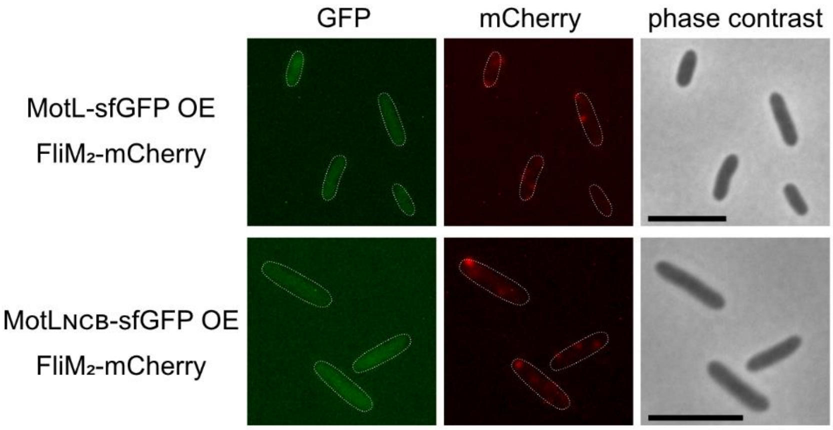

Supplemental Figure 4: Localization of MotL. Shown are micrographs of overproduced (OE) sfGFP-tagged MotL (first panel) in cells in which also the C-ring protein $FliM_2$ of the lateral flagella is C-terminally labelled with mCherry (middle panel). The phase contrast images of the cells are in the right panel. While lateral C-rings are readily detected, no distinct localization of MotL was detected. The scale bar equals 5 µm.

ADDITIONAL REFERENCES

Bubendorfer, S., Held, S., Windel, N., Paulick, A., Klingl, A., and Thormann, K. M. (2012). Specificity of motor components in the dual flagellar system of *Shewanella putrefaciens* CN-32. *Mol. Microbiol.* 83, 335–350. doi: 10.1111/j.1365-2958.2011.07934.x

Bubendorfer, S., Koltai, M., Rossmann, F., Sourjik, V., and Thormann, K. M. (2014). Secondary bacterial flagellar system improves bacterial spreading by increasing the directional persistence of swimming. *Proc. Natl. Acad. Sci. U. S. A.* 111, 11485–11490. doi: 10.1073/pnas.1405820111

Fredrickson, J. K., Zachara, J. M., Kennedy, D. W., Dong, H., Onstott, T. C., Hinman, N. W., et al. (1998). Biogenic iron mineralization accompanying the dissimilatory reduction of hydrous ferric oxide by a groundwater bacterium. *Geochim. Cosmochim. Acta* 62, 3239–3257. doi: 10.1016/S0016-7037(98)00243-9

Karimova, G., Pidoux, J., Ullmann, A., and Ladant, D. (1998). A bacterial two-hybrid system based on a reconstituted signal transduction pathway. *Proc. Natl. Acad. Sc.i U. S. A.* 95, 5752–5756. doi: 10.1073/pnas.95.10.5752

Karimova, G., Ullmann, A., and Ladant, D. (2002). A bacterial two-hybrid system based on Cyclic AMP signalling cascade. *A Molecular Cloning Manual*, 447–488.

Lassak, J., Henche, A.-L., Binnenkade, L., and Thormann, K. M. (2010). ArcS, the cognate sensor kinase in an atypical Arc system of Shewanella oneidensis MR-1. *Appl. Environ. Microbiol.* 76, 3263–3274. doi: 10.1128/AEM.00512-10

Miller, V. L., and Mekalanos, J. J. (1988). A novel suicide vector and its use in construction of insertion mutations: osmoregulation of outer membrane proteins and virulence determinants in *Vibrio cholerae* requires *toxR*. *J. Bacteriol.* 170, 2575–2583. doi: 10.1128/jb.170.6.2575-2583.1988

Pédelacq, J.-D., Cabantous, S., Tran, T., Terwilliger, T. C., and Waldo, G. S. (2006). Engineering and characterization of a superfolder green fluorescent protein. *Nat. Biotechnol.* 24, 79–88. doi: 10.1038/nbt1172

Peränen, J., Rikkonen, M., Hyvönen, M., and Kääriäinen, L. (1996). T7 vectors with modified T7*lac* promoter for expression of proteins in *Escherichia coli*. *Anal. Biochem.* 236, 371–373. doi: 10.1006/abio.1996.0187

Rossmann, F., Brenzinger, S., Knauer, C., Dörrich, A. K., Bubendorfer, S., Ruppert, U., et al. (2015). The role of FlhF and HubP as polar landmark proteins in *Shewanella putrefaciens* CN-32. *Mol. Microbiol.* 98, 727–742. doi: 10.1111/mmi.13152

Schuhmacher, J. S., Rossmann, F., Dempwolff, F., Knauer, C., Altegoer, F., Steinchen, W., et al. (2015). MinD-like ATPase FlhG effects location and number of bacterial flagella during C-ring assembly. *Proc. Natl. Acad. Sci. U. S. A.* 112, 3092–3097. doi: 10.1073/pnas.1419388112
